# Medical Opinion and Movement

**Published:** 1911-01-21

**Authors:** 


### Medical Opinion and Movement.

### Treatment of Acne.

Acne will yield to persistent and judicious medical treatment, though it is admittedly one of the most stubborn of cutaneous affections. The routine recommended by Dr. Cocks in the *Medical Record* is as follows. Strict rules are to be laid down and observed as to the avoidance of cakes, pies, pastries, fish, and all salt meats. Ice-creams and similar delicacies are strictly forbidden, and so is eating between meals. Indolent and obese patients must be forced to take outdoor exercise. Their general sluggishness and constipation are treated by a mixture of potassium acetate, 8 grains; fluid extract of cascara, 20 minims; and fluid extract of rumex, 30 minims, given in half a glassful of cold water half an hour before meals. Anæmic patients receive extra diet in the way of eggs, milk, cream, rice, soup, and meats; they also take a mixture containing citrate of iron, strychnine, and magnesium sulphate. If comedones or pustules are present, a rapid impression can be made with green soap, 1 drachm; resorcin, 1 drachm; salicylic acid, 5 grains; and rose-water ointment, 2 ounces. This is applied at night and washed off in the morning. It is repeated until a fair amount of desquamation has been obtained. Another plan is to express all comedones and incise all pustules, touching the bases with hydrogen peroxide followed by carbolic acid. The outcome, Dr. Cocks says, is primary union without scarring. Constant cleanliness and slight friction with the towel are also of service in keeping the skin active and healthy.

### Sarcoma of the Clavicle.

The signs and treatment of this uncommon disease, as established by a study of sixty-three published and unpublished cases, are discussed by Dr. W. B. Coley in the *Annals of Surgery*. Several of the patients came under his own personal observation. As elsewhere, early diagnosis is of course the only sure basis for successful treatment; and to periosteal sarcomata, as being so frightfully malignant, the remark especially applies. Fortunately, the clavicle is so close to the surface that difficulty in making the diagnosis is, and ought to be, slight. In most cases there is a history of antecedent injury, sometimes by a direct blow, sometimes by a wrench or strain. Seldom is there any clear evidence of sarcoma beginning at the site of fracture in a healthy clavicle. As a rule swelling appears after the injury; it may partially subside, to be replaced a few weeks later by the enlargement due to the growth. Pain is usually absent, and the skin and glands are not involved in the early stages. X-ray examination is of great help in diagnosis. Tuberculosis and syphilis are the two conditions from which a differential diagnosis has to be made. Dr. Coley says the clinical signs are sufficient without microscopical examination; a rapidly growing tumour in this situation should be removed without any delay. Out of the whole series of sixty-three cases but six of successful cure are found; this is chiefly due to deferring operation until too late. The prophylactic treatment with bacillary toxins associated with this surgeon's name is strongly recommended as soon as the primary operation wound is healed. This, the intimate association of injury with the condition, and the need for instant diagnosis and treatment, are the three main heads of the author's discourse on this unusual growth.

### Bevan's Operation for Undescended Testis.

A strong plea for the adoption of this little-known operation is put forward by Dr. A. V. Moschcowitz in the *Annals of Surgery*. For long it was believed that the spermatic artery is an end artery and constitutes the sole supply of the testis. It has, however, been shown that this is not strictly accurate, and that the artery to the vas supplies the testis also; moreover, this source of supply is by no means contemptible, for when the spermatic artery is ligatured it can replace it and save the organ from necrosis. It is by utilising this research that Bevan has devised a method of bringing down the undescended testis, which is described as highly successful. The essential part of this method is the freeing and division of the spermatic vessels, which are the real obstacles to the bringing down of the testis. The vas deferens is always much longer than the vessels, and when once these are ligatured and divided there is no need to anchor the testis to the bottom of the scrotum. Great care must be taken never to handle the vas roughly or to touch it with forceps, lest thrombosis of the all-important tiny artery should ensue. The sac of any hernia which may exist is cut away and the inguinal canal sutured as in any of the recognised radical cures; the vas is not transplanted. Two or three days after operation there is often a good deal of swelling and œdema of the testis, which, however, soon passes off under ordinary treatment. The author finds that a complete cure results; there is no recurrence of hernia, and a freely movable testis remains in the bottom of the scrotum. He does not claim that spermatogenesis, so often lacking in ectopic testicles, is restored; but it is worth noting that a patient in whom this operation was done bilaterally had at least perfect sexual potency.

### The Death-rate from Puerperal Sepsis.

Several times in recent years leaders of the obstetrical branch of the profession have called attention (Sir F. Champneys and Dr. Dakin, for example) to the fact that the mortality ratio from puerperal sepsis has been very little, if at all, diminished in the country at large by the introduction of antiseptic midwifery. The death-rate in institutions has, it is true, been reduced to a level no higher than that outside them; but the general rate for the community at large is not diminishing as it should do. The same serious lesson is learnt from figures quoted by Dr. Haultain in the *Edinburgh Medical Journal*, from which it appears that whereas in England the death-rate from sepsis per 1,000 deliveries is 1.4, in Scotland it is 1.7. Yet in Italy it is but 0.9, in Sweden 0.8, and in Holland 0.7. Nor can the explanation be the better notification of cases, for the total death-rate in the puerperium is just as high now as it was fifty years ago, both in Scotland and in the capital city, Edinburgh, though in England there is a distinct decline. A very remarkable point shown by the statistics is the rapidly increasing devastation caused by eclampsia. In Edinburgh lying-in hospitals cases of this disorder are six times as numerous as in English and Irish institutions; and the case mortality is two and a half times as great. Thus the death-rate from convulsions per 1000 deliveries is fifteen times as great in Scotland as in the rest of the kingdom. The author thinks it possible that the replacement of a farinaceous diet by one much richer in nitrogenous ingredients may explain this; but then the English never did live on porridge and potatoes, and, according to this argument, should have suffered far more than the Scotch and Irish from eclampsia in times past. Whatever the cause, the fact is most alarming, and well deserving of the attention which Dr. Haultain has drawn to it.

### Carcinoma of the Tongue in Edinburgh.

Some rather curious facts about lingual carcinoma are noted by Professor Caird in a contribution to the *Edinburgh Medical Journal*. Thus in the sixty cases analysed, only four times was a history of syphilis acknowledged; and in no case was there any actual luetic manifestation, save leucoplakia, which was found thrice only. With regard to alcohol, three patients were abstainers, but at least fourteen drank to excess. Not a single male was a non-smoker. There was no case in which heredity could be traced; indeed, only five patients had a family history of malignant disease in any situation. Twice the microscope failed to confirm the diagnosis, which was made first clinically and afterwards by the course and termination of the malady. Curiously, both these cases occurred in women, of whom there were but six in the series; in one of these operation was advised against on the strength of two consecutive negative reports, but the patient eventually came back with inoperable extension of the growth. The only notable feature of the author's operative technique is his employment of local anæsthesia for the first stage of the Butlin operation; that is, for the proceeding usually known as Whitehead's operation. The infiltration of the anæsthetic solution opens up connective tissue planes, thus facilitating the recognition of anatomical details and of blood-vessels, as well as rendering a general anæsthetic unnecessary. Even if the mandible has to be divided, this method of local anæsthesia will suffice; and it renders tracheotomy unnecessary. Whether all patients, especially in private practice, will be callous enough to undergo so severe a mutilation without a general anæsthetic may perhaps be doubted.

### Exophthalmic Goitre and Constipation.

One of the oldest and still the most fundamental indications of medical treatment in all manner of ailments and maladies is "keep the bowels open." Dr. W. Ebstein, of Goettinger, has raised this medical platitude to a primary principle of treatment in cases of exophthalmic goître. He points out that this disease is frequently accompanied by chronic constipation, and he finds that in such cases efficacious treatment of the constipation not only diminishes the symptoms of the diseases, but may cause them completely to disappear. The hypothesis put forward is that the absorption of toxins consequent upon the intestinal stasis provoke the phenomena of thyroidism in a patient of that disposition. However this may be, the author gives details of several cases of well-marked exophthalmic goître in which daily injections of oil followed by enemata of saline solution have been followed by complete disappearance of all the symptoms of the disease. In three cases the efficacy of the treatment was especially well marked, inasmuch as the patients had been treated previously for some time by all manner of medicaments without any alleviation of the symptoms. In some cases the constipation alternates with diarrhœa, but the author regards such diarrhœic conditions as resulting from intestinal irritation due to the stasis of fæcal matter in the intestines, and he finds that the condition similarly yields to oil injections.

### Duration of Fluids in the Stomach.

Dr. Kastle has devised a method of examining the stomach with the $x$-rays to determine the length of time that fluids remain there before complete evacuation. An account of the work is given in the *Münchener Medizinische Wochenschrift*. For this purpose he uses "floating capsules" of gelatin with a covering of indiarubber in order to protect them against the action of the gastric juice and containing bismuth carbonate, oxide of thorium, or oxide of zirconium. The subject being in a fasting condition is given a few grammes of oxide of zirconium in cachets in order to determine by radioscopic examination the lower limits of the stomach. The fluid with which one intends to experiment is then given together with three capsules of 6 to 10 millimetres diameter. These capsules float on the surface of the liquid and approach the lower margin of the stomach in proportion as the fluid is evacuated from the stomach. In this way the author has been able to determine the length of time that different fluids remain in the stomach of healthy subjects. Thus he finds that an hour and a half after the absorption of 250 c.c. of water there is only a small amount left, which completely disappears, together with the capsules, in the course of another twenty minutes. The same quantity of warm milk is almost entirely evacuated at the end of two hours and a half; 250 c.c. of cocoa with milk remains two hours and three-quarter, and the same quantity of tea slightly sweetened, but without milk, remains only an hour and a half. On the other hand, 250 c.c. of red wine is not completely evacuated at the end of two hours twenty-five minutes.

### Injections of Camphorated Oil.

Höhne and Pfannenstiel first conceived the idea of employing camphorated-oil injections into the peritoneal cavity as a prophylactic measure against peritonitis in cases of laparotomy for severe infective conditions. The treatment was based upon certain experiments upon animals. They found that if an intra-peritoneal injection of oil were made some days before introducing a very virulent culture of bacillus coli into the peritoneal cavity an inflammatory reaction was produced which had the effect of opposing the absorption of the inoculated microbes and enabled the animals successfully to resist infection. These facts have already been referred to about a year ago in these columns. Since then the method has been taken up by Dr. K. Kolb, of Bâle. In the course of a year 172 laparotomies have been performed, and in seventy-nine of these intra-peritoneal injections of sterilised camphorated oil have been administered. All the cases were gynæcological and included conditions of pyo-ovaritis, tubular gestations, tuberculosis of the genital organs, cancer of the uterus, etc. Instead of injecting the oil two or three days before operation, according to the method of Höhne and Pfannenstiel, Kolb introduced the oil at the time of the operation, or, if there were a glass drainage-tube, immediately after the operation, to the extent of 50 c.c. The strength of the camphorated oil was 10 per cent., the same as that used for subcutaneous injections in cases of pneumonia. Considering only those cases in which there was definite infection of the peritoneum during the operation, such as the penetration of pus from an ovarian cyst into the peritoneal cavity, the author had nine deaths out of fifty-three such cases, and only one of them was due to peritonitis, giving a mortality of 1.8 per cent. for peritonitis. In four cases there were cutaneous abscesses showing the virulence of the infection.

### Rectal Injections of Paraffin.

Reference was recently made in these columns to the treatment of intestinal stasis and chronic constipation by the administration of liquid paraffin and vaseline by the mouth. Dr. Lipowski, of Bromberg, advocates rectal injections of paraffin for the same purpose. He points out that it is necessary to ascertain that the patient is really suffering from chronic constipation characterised by hard stools and scybalæ often covered with mucus. Elderly people frequently complain of constipation, although they may be having one or two evacuations a day. The treatment is not indicated in nervous and hysterical patients or in cases in which there is some mechanical obstruction. The author used a mixture consisting of 8 parts of solid paraffin and 1 part of vaseline. The mixture is first warmed and 200 c.c. injected by means of an ordinary syringe and a tube, which should also be previously warmed to prevent the mixture setting. The tube should be introduced into the rectum to a depth of 10 cm. to 15 cm. The injection should take place at bedtime, and if there is no spontaneous evacuation in the morning, resort should be had to a saline enema. At the end of a week or so the amount injected may be reduced to 100 c.c., and then a week later the injections should be given every other day, a saline enema being administered on the alternate days if there is no natural evacuation. In this way the author claims to have successfully treated several cases of chronic constipation in the course of a few weeks. Any return of hard stools should be immediately treated by a paraffin injection.

### Gangrene of the Arm after Ergot Poisoning.

At a recent meeting of the Société Médicale des Hôpitaux, Drs. Dufour and Huber showed a patient who had been admitted to the Maternité Hospital in a state of coma. The existence of a five months' pregnancy gave rise to the idea that the patient was eclamptic, but bleeding gave no relief to the agitation which followed coma, and was accompanied by hyperthermia. It was then noticed that the right arm was paralysed and cold. The general condition improved after a time, but the limb remained cold, black phlyctenules appeared, and the axillary pulse could no longer be felt. In the presence of these signs of gangrene by arteritis it was found necessary to amputate the arm, upon which the existence of a clot extending to the axillary artery was noticed. The bloodless and grey anterior muscles were cut away also. For a long time the general condition of the patient was very bad, fever, multiple abscesses, parotitis, otitis, mastoiditis, and finally a slight degree of phlebitis being present. With some difficulty the authors were able to extract from the patient the information that she had taken ergot for the purpose of inducing abortion. In her case the drug had produced two types of ergotism, the convulsive and the gangrenous. Examples of this kind were met with more commonly formerly than at the present day.

### Menthol Poisoning in Infants.

Of late several cases have been reported in which, as the result of ingestion of menthol and its derivatives by young children dangerous symptoms arose. Armand-Delille, of Paris, recorded a case of spasm of the glottis in an infant after nasal instillation of oil and menthol. Koch, of Fribourg, has instanced a similar result after the use of corifine, a menthol derivative; and Kilian has published a case of dangerous syncope following administration of oil and menthol. In Spain, Dr. Guerra y Estapé has recorded another analogous case. A recent number of *Clinica y Laboratorio* contains a report by Dr. R. H. Alcorta of the case of his own child, aged one month, who was treated for coryza, which interfered with suckling, by a nasal instillation of 1-per-cent. menthol in oil. Respiration stopped suddenly, the child's face became pale, its lips cyanosed, its mouth wide open, and its pulse imperceptible. The child was in a state of imminent asphyxia, which necessitated douching with cold water, artificial respiration, tickling of the epiglottis, and other manœuvres for a considerable period of time before relief could be obtained. There would thus seem to be sufficient evidence on record to prohibit the use of menthol where young children are concerned.

